# Lag correction model and ghosting analysis for an indirect‐conversion flat‐panel imager

**DOI:** 10.1120/jacmp.v8i3.2483

**Published:** 2007-07-23

**Authors:** Noor Mail, Peter O'Brien, Geordi Pang

**Affiliations:** ^1^ Toronto Sunnybrook Regional Cancer Centre University of Toronto Toronto Ontario Canada

**Keywords:** cone‐beam digital tomosynthesis, cone‐beam computed tomography, image lag, ghosting, flat‐panel imager

## Abstract

Cone‐beam digital tomosynthesis (CBDT) is a new approach that was recently proposed for rapid tomographic imaging of soft‐tissue targets in the radiotherapy treatment room. One of the potential problems in implementing CBDT using, for example, megavoltage (MV) X rays is the possibility of artifacts caused by image lag and ghosting of the X‐ray detector used. In the present work, we developed a model to correct for image lag with indirect‐conversion flat‐panel imagers (FPIs) used for MV‐CBDT. This model is based on measurement and analysis of image lag in an indirect‐conversion FPI irradiated with a 6‐MV X‐ray beam. Our results demonstrated that image lag is amenable to correction. In addition, we measured the ghosting effect for an indirect‐conversion FPI and found it to be insignificant.

PACS numbers: 87.53.Oq, 87.57.Ce

## I. INTRODUCTION

Motion of soft‐tissue targets, such as that of a lung tumor, is one of the main concerns in high‐precision radiation therapy.^(^ ^1^ ^–^ ^3^ ^)^ Cone‐beam digital tomosynthesis (CBDT) is a new approach that was recently proposed for rapid tomographic imaging of soft‐tissue targets in the radiotherapy treatment room.^(^ ^4^ ^–^ ^6^ ^)^ Because it utilizes partial scans (typically in the range 20 – 60 degrees of gantry arc), CBDT can be thought of as limited‐angle cone‐beam computed tomography (CBCT).^(^ ^7^ ^–^ ^9^ ^)^ The main advantage of CBDT is that it is faster than CBCT, and thus it can potentially be used for image‐guided lung treatment (for example). In a deep inspiration breath hold (DIBH) technique, a CBDT image acquisition can be accomplished during the patient's breath hold—for instance, 5 seconds. The CBDT images can then be used to guide the treatment during DIBH. In addition, CBDT can be extended to the time domain (that is, four‐dimensional CBDT) to replace four‐dimensional CBCT^(^ ^10^ ^–^ ^13^ ^)^ for image‐guided respiratory‐gated lung treatment.^(^ ^4^ ^,^ ^14^ ^)^

One of the potential problems in implementing CBDT is the possible artifact caused by image lag and ghosting in the X‐ray detector used. Image lag is a residual signal present in image frames subsequent to the frame in which the residual signal was generated. Ghosting refers to the change of detector pixel sensitivity (to X‐rays) recause of previous exposures of the detector. Image lag and ghosting have been found in both direct‐ and indirect‐conversion flat‐panel imagers (FPIs).^(^ ^15^ ^–^ ^20^ ^)^ Although the exact mechanism of image lag or ghosting depends on the design of the particular system, charge trapping and the release of trapped charge in detector elements are respectively considered to be the main sources of ghosting and image lag in FPIs.^(^ ^15^ ^,^ ^19^ ^)^

The severity of ghosting in FPIs is determined mainly by the total accumulated charge trapped in detector elements; the severity of image lag is determined mainly by the rate of release of previously trapped charge. In general, faster release of previously trapped charge results in less accumulated charge being trapped. Thus, a FPI with severe image lag usually has little ghosting, and vise versa.

The presence of image lag or ghosting as low as approximately 1% can cause significant image artifacts during megavoltage (MV) X‐ray imaging, because the subject contrast for MV X ray is, in general, very low (a few percent, approximately). Most current FPIs used for MV X‐ray imaging are based on indirect conversion, in which image lag instead of ghosting is most significant.^(^ ^15^ ^,^ ^16^ ^)^

Image lag is not necessarily a concern for radiographic imaging of a *static* target (for example, the bony anatomy of a patient) with a *fixed* gantry angle—as in conventional portal imaging.^(21)^ However, it is a concern for MV‐CBDT, in which *fluoroscopic* imaging is required, given that either the target or the gantry is moving during image acquisition. Previous work in this area has focused on quantifying image lag and ghosting and their effects on image quality.^(^ ^15^ ^–^ ^20^ ^)^ However, little work has been done to correct for the lag and ghosting effects, especially for MV X‐ray imaging.

In the present work, we developed a model to correct for image lag in indirect‐conversion FPIs used in MV‐CBDT. We based this model on measurement and analysis of image lag in an indirect‐conversion FPI at 6 MV. Our results demonstrated that image lag is amenable to correction. In addition, we measured the ghosting effect for indirect‐conversion FPI and found it to be insignificant.

## II. MATERIALS AND METHODS

### A. The indirect‐conversion FPI

The X‐ray detector used in the present work is an indirect‐conversion FPI (RID 1640: PerkinElmer, Waltham, MA). The active imaging area of the detector is 40×40 cm^2^ and the total number of pixels is 1024×1024 with each 400×400 mm^2^. The detector consists of a Cu plate and phosphor screen (Kodak Lanex fast: Carestream Health, Rochester, NY) coupled to an active readout matrix. The screen is used to convert X‐ray energies into light, which is absorbed by photodiodes integrated into the active‐matrix flat‐panel array and stored as charge on the capacitance of the photodiodes.^(22)^ The latent charge image is then read out using the active components on the active matrix, switching them using thin film transistors. The detector can be operated continuously with a frame rate ranging from 0.1 fps to 3.5 fps. The range of ambient temperature of operation is 15±C to 35±C.

The FPI was connected to a computer running image acquisition software (HIS version 2.3: PerkinElmer). In practice, two calibration files, one for offset and a second for gain, are established at the beginning of each experiment. The offset image is acquired to account for the dark current of the panel when no radiation is present. The gain correction or bright image is taken with a dose of 1 cGy (dose to water at detector surface) to allow for determination of differences in pixel sensitivity.

### B. Lag measurements

During the lag measurements, the detector was placed at a source‐to‐detector distance of 135 cm from the 6‐MV X‐ray source of a linear accelerator (LINAC) with no object between the source and the detector. A radiation field (with a field size of 40×40 cm^2^ at the detector surface) was first delivered at frame 0, and the signals in subsequent dark frames (no X rays) were measured. These subsequent frames (frames 1, 2, and so on) contain signal attributable only to lag. The amount of lag in the *n*th frame $\left(L_{n}\right)$ was calculated based on the formula

(1) $$L_{n}=\left(\frac{I_{n}-B}{I_{0}-B}\right)\times 100\;,$$

where $I_{n}$ and $I_{0}$ are the mean detector signal over a region of interest containing 1000 pixels in, respectively, the *n*th frame and the 0th frame, and *B* is the dark current determined from the offset images. The image lag was measured as a function of radiation dose (delivered at frame 0) over the range 0 – 2 cGy and the frame rate. These measurements were repeated four times, and the results were averaged.

### C. Lag correction model

A model was developed to permit calculation of the lag in any frame (see subsection III.B). To demonstrate the effectiveness of the lag correction model, projection images of a moving lead aperture were obtained before and after the lag correction. The lead aperture was placed between the LINAC and the FPI (100 cm from the LINAC and 30 cm from the FPI). The aperture was 4 cm in thickness, with a hole 2.5 cm in diameter in the middle. The aperture was mounted on a high‐precision translation stage and was moved perpendicular to the incident X‐ray beam. Approximately 20 images were acquired at a frame rate of 3.5 fps. The aperture was stationary during the first 7.5 frames and started moving in the second half of the 8th frame. The speed of the aperture was set at 30 cm/s.

**Figure 1:**
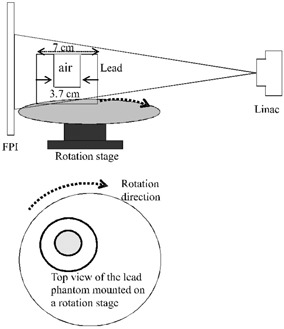
Schematic of the cone‐beam digital tomosynthesis (CBDT) setup designed for studying the image lag effect on the reconstructed CBDT image. The object is a hollow lead cylinder. FPI=flat‐panel imager.

Fig. 1 shows the experimental setup for investigating the effect of image lag and its correction in reconstructed CBDT images. A hollow lead cylinder of outer diameter 7 cm and inner diameter 3.7 cm was used. This lead cylinder was placed on a high‐precision rotation stage whose axis of rotation was placed at a distance of 100 cm from the LINAC source and 35 cm from the FPI. All components, including the rotation stage, were computer‐controlled, providing synchrony between X‐ray exposure, object rotation, and FPI readout. The FPI was operated with a maximum frame rate of 3.5 fps.

This CBDT system was designed to acquire 30 projections (that is, a α=15 degrees^(14)^) in less than 15 seconds using the FPI's continual image acquisition mode. The rotation motion of the stage was adjusted to be slow enough (2 degrees/s) to minimize motion artifact. Among the 30 resulting projection images, 29 (2 – 30) were corrected for image lag effects by subtracting the previous projections weighted by the magnitude of image lag in those projections. Because of noise accumulation, the previous projections subtracted were limited to 10 (in fact, the lag signal is insignificant after 10 frames). Finally, after correction, a CBDT reconstruction code based on a filtered backprojection algorithm (Siemens Medical Systems, Concord, CA) used the 30 projections to reconstruct a planar image; the image was then compared to an image obtained without lag correction.

### D. Ghosting measurements

In the ghosting experiment, the indirect‐conversion FPI was powered off for 24 hours before each measurement. After re‐application of power, the dark current was allowed to stabilize for 2 hours before the first X‐ray exposure. Offset and gain corrections were made immediately before the ghosting exposure. All measurements were made at 6 MV.

The experimental setup for the ghosting measurements was similar to that used for image lag, except that a computer‐controlled rotation stage was used to place a lead aperture 10 cm in thickness in the X‐ray beam during the ghosting exposure and then to move it out of the beam immediately after the exposure. This setup induced a pixel sensitivity change in the region where the ghosting exposure was delivered. The doses used in the ghosting exposures were in the range 10 – 2000 cGy.

To measure the pixel sensitivity change (that is, the ghosting effect), the FPI was operated at 3.5 fps, and 20 frames were acquired either immediately or 20 minutes after the ghosting exposure. To estimate the dark signal (including the lag from the ghosting exposure), the first 10 frames were acquired without radiation. The last 10 frames were acquired with radiation (testing) exposures. The detector saturation for all testing exposures (approximately 1 cGy) was 40%. The ghosting effect was then determined as

(2) $$g_{n}=\frac{S_{A}}{S_{B}}\;,$$

where $S_{A}$ is the averaged pixel value of the ghost‐induced region A, and $S_{B}$ is the averaged pixel value of the ghost‐free region B in the testing images (after correction of the dark signal in both regions). The measured sensitivity $\left(g_{n}\right)$ contains no lag from the ghosting exposure, but may contain some lag from the testing exposures. However, the lag attributable to testing exposures is present at about the same magnitude in regions A and B alike. As a result, the effect of the lag on the sensitivity measurement is approximately $L_{1}^{\;\;2}<0.01\%$. The sensitivity $\left(g_{n}\right)$ was measured as a function of ghosting dose.

## III. RESULTS AND DISCUSSION

### A. Image lag

**Figure 2:**
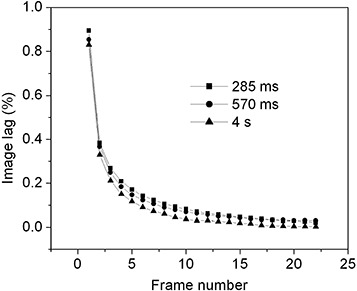
Image lag measured at 6 MV as a function of frame number (equation 1) for three different readout times.

Fig. 2 shows three sets of lag data as a function of frame number for three different frame times (inversely proportional to the frame rate). At frame times 285 ms and 570 ms, lag is nearly identical, but at a very long frame time (4 s), the lag signal as a function of frame number decays slightly more because of a small dependence of lag on time after exposure. This observation suggests that lag in the *n*th frame depends primarily on the number of frames (reads) and weakly on time since the exposure. Fig. 2 further illustrates that the magnitude of image lag decreases rapidly during the first few frames and then more slowly in subsequent frames.

**Figure 3:**
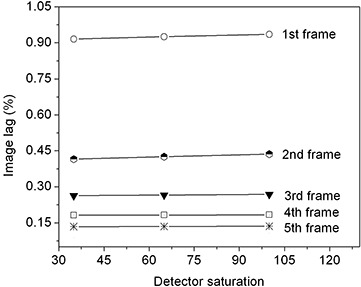
Image lag as a function of dose (expressed by detector saturation) for various frames. The measurements were performed at 6 MV with a frame rate of 284 ms.

We also examined image lag as a function of dose to the FPI. In Fig. 3, lag is plotted as a function of dose expressed by detector saturation for various frames. For all frames, the image lag does not change significantly as a function of detector dose (100% saturation of the detector is reached at a dose of approximately 2 cGy).

### B. Lag correction model

**Figure 4:**
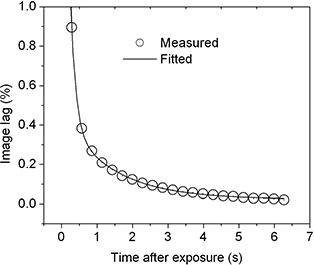
Theoretical fit to the lag data measured at frame rate of 570 ms.

We found that the measured data can be fitted using the formula

(3) $$L_{n}=C_{o}+C_{1}\exp\left(-\frac{n\tau }{P_{1}}\right)+C_{2}\exp\left(-\frac{n\tau }{P_{2}}\right),$$

where $C_{0},C_{1},\;\text{and}\;C_{2}$ are coefficients that depend weakly on the frame rate, and $P_{1}\;\text{and}\;P_{2}$ are the two decay constants (for rapid decay and slow decay respectively), which depend strongly on the frame rate, but not on the dose to the detector. We note that a similar form was used by Siewerdsen and Jaffray.^(16)^ Fig. 4 shows the use of equation 3 to produce one theoretical fit to image lag data. Fits were similar for all frame rates. Table 1 lists the fitted coefficients and parameters for frame times 285 ms and 570 ms.

**Table 1:** Values of the fitted coefficients $\left(C_{0},\;C_{1},\;C_{2}\right)$ and parameters $\left(P_{1},\;P_{2}\right)$ for two cases: τ=285 ms and 570 ms

τ	$C_{0}$	$C_{1}$	$C_{2}$	$P_{1}$	$P_{2}$
285 ms	0.02329±0.00242	3.80198±0.35208	0.14333±0.0079	0.43035±0.0161	1.38331±0.0613
570 ms	0.0242±0.0013	2.919±0.1647	0.3191±0.0124	0.3468±0.0125	2.6817±0.0969

During fluoroscopic imaging, the *n*th frame may contain the contribution of lag from all previous frames. Our lag correction model determined “lag‐free” signal in the *n*th frame (that is, the signal if lag were not present) using the equation

(4) $$I_{nc}=I_{n}-L_{1}I_{n-1}-L_{2}I_{n-2}-L_{3}I_{n-3}.....L_{m}I_{n-m}=I_{n}-\sum_{m=1}^{n}L_{m}I_{n-m},$$

in which frame *n* is variously frame 1, frame 2, and so on, and $I_{n}$ is the measured total signal (including lag) in frame *n*. Based on equation 3, equation 4 can be rewritten as

(5) $$I_{nc}=I_{n}-\sum_{m=1}^{n}I_{n-m}\left(C_{o}+C_{1}\exp\left(-\frac{m\tau }{P_{1}}\right)+C_{2}\exp\left(-\frac{m\tau }{P_{2}}\right)\right)\cdot$$

### C. Demonstration of lag correction for projection images

**Figure 5:**
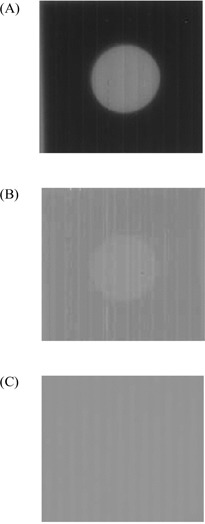
(A) Projection image of a lead aperture immediately before motion (7th frame). (B) Image lag obtained immediately after the lead aperture was moved out of the original location (9th frame). (C) Image after lag correction for the 9th frame.

**Figure 6:**
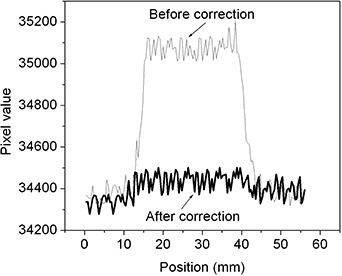
Intensity profiles along a horizontal line through the center of the images from Fig. 5(B,C).

Fig. 5(A) shows the 7th frame image obtained (immediately before the aperture started to move). Fig. 5(B,C) shows the image of the 9th frame before and after lag correction. Fig. 6 shows the intensity profiles along a horizontal line through the center of the image before and after lag correction. The intensity profile of the image before lag correction shows a clear lag effect of approximately 2% of the total signal. The corrected image profile shows clear improvement (reduction of lag artifact), with more than 80% of the lag artifact successfully corrected.

### D. Lag correction to CBDT images

**Figure 7:**
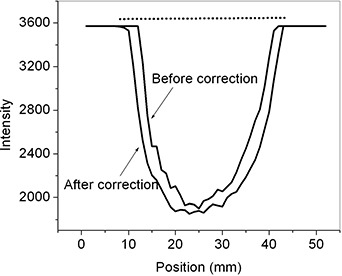
Intensity profiles of the reconstructed images (along a line crossing the center of the hollow lead cylinder) before and after lag correction. The dotted straight line represents the real size of the object (hole).

Fig. 7 shows the intensity profiles of the CBDT reconstructed images (along a horizontal line crossing the center of the air hole, shown at the bottom in Fig. 1) before and after lag correction. Image contrast can be seen to be slightly improved after lag correction. The object size measured from the intensity profile of the reconstructed image before correction is smaller than the object's (hole's) real size. This is the result of the lag artifact in each projection image. After lag correction, the size matching to the hole is much improved.

### E. Ghosting

**Figure 8:**
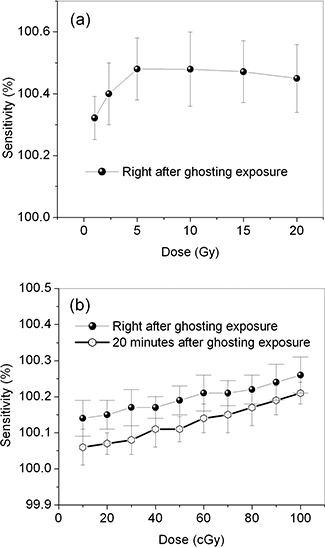
Change in sensitivity as a function of dose (induced ghost) at 6 MV with (a) high dose, and (b) low dose.

Fig. 8(a) shows the sensitivity of the ghost‐exposed region of the FPI, plotted as a function of ghosting exposure at 6 MV. With ghosting doses up to 5 Gy, an increase in sensitivity is evident. Within experimental error, the detector sensitivity is almost constant or decreases slightly with ghosting doses from 5 Gy to 20 Gy.

Fig. 8(b) shows two sets of sensitivity data plotted as a function of low ghosting exposure dose: one set taken immediately after the ghosting exposure, and the other taken after a 20‐minute pause. The ghosting effect on sensitivity was reduced after a pause of 20 minutes. For this FPI, the pixel sensitivity change induced by ghosting at doses that would be used for clinical imaging (up to 10 cGy) is not significant (approximately 0.1%) as compared with the signal caused by image lag (approximately 1%). To reduce the ghosting effect on CBCT, CBDT, and radiographic images, offset and gain corrections can be made (after each patient is imaged, for example) to renormalize pixel sensitivity values and minimize this effect.

## IV. CONCLUSIONS

We investigated image lag and ghosting for an indirect‐conversion FPI at 6 MV. We found that, in the FPI, image lag is more significant than ghosting and that it can cause significant artifacts in MV‐CBDT. We developed a model to correct for the image lag. Our results demonstrated that image lag is amenable to correction, and our correction method will be useful for reducing lag artifacts in MV‐CBDT.

## ACKNOWLEDGMENTS

Helpful discussions and suggestions from Dr. John Rowlands are gratefully acknowledged. This work was supported by Siemens Medical Systems, Inc., Concord, California, U.S.A.

## References

[1] Langen KM , Jones DT . Organ motion and its management. Int J Radiat Oncol Biol Phys. 2001; 50 (1): 265–278. 11316572 10.1016/s0360-3016(01)01453-5

[2] Seppenwoolde Y , Shirato H , Kitamura K , et al. Precise and real‐time measurement of 3D tumor motion in lung due to breathing and heartbeat, measured during radiotherapy. Int J Radiat Oncol Biol Phys. 2002; 53 (4): 822–834. 12095547 10.1016/s0360-3016(02)02803-1

[3] Sixel KE , Ruschin M , Tirona R , Cheung PC . Digital fluoroscopy to quantify lung tumor motion: potential for patient‐specific planning target volumes. Int J Radiat Oncol Biol Phys. 2003; 57 (3): 717–723. 14529776 10.1016/s0360-3016(03)00713-2

[4] Pang G , Au P , O'Brien P , Bani‐Hashemi A , Svatos M , Rowlands JA . Cone beam digital tomosynthesis (CBDT): an alternative to cone beam computed tomography (CBCT) for image‐guided radiation therapy [Abstract]. Med Phys. 2005; 32: 2126.

[5] Svatos M , Pang G , Gangadharan B , et al. 4D cone beam digital tomosynthesis (CBDT) and digitally reconstructed tomograms (DRTs) for improved image guidance of lung radiotherapy [Abstract]. Med Phys. 2005; 32: 2161.

[6] Godfrey DJ , Yin FF , Oldham M , Yoo S , Willett C . Digital tomosynthesis with an on‐board kilovoltage imaging device. Int J Radiat Oncol Biol Phys. 2006; 65 (1): 8–15. 16618573 10.1016/j.ijrobp.2006.01.025

[7] Jaffray DA , Siewerdsen JH , Wong JW , Martinez AA . Flat‐panel cone‐beam computed tomography for image‐guided radiation therapy. Int J Radiat Oncol Biol Phys. 2002; 53 (5): 1337–1349. 12128137 10.1016/s0360-3016(02)02884-5

[8] Seppi EJ , Munro P , Johnsen SW , et al. Megavoltage cone‐beam computed tomography using a high‐efficiency image receptor. Int J Radiat Oncol Biol Phys. 2003; 55 (3): 793–803. 12573767 10.1016/s0360-3016(02)04155-x

[9] Pouliot J , Bani‐Hashemi A , Chen J , et al. Low‐dose megavoltage cone‐beam CT for radiation therapy. Int J Radiat Oncol Biol Phys. 2005; 61 (2): 552–560. 15736320 10.1016/j.ijrobp.2004.10.011

[10] Sonke JJ , Zijp L , Remeijer P , van Herk M . Respiratory correlated cone beam CT. Med Phys. 2005; 32 (4): 1176–1186. 15895601 10.1118/1.1869074

[11] Dietrich L , Jetter S , Tucking T , Nill S , Oelfke U . LINAC‐integrated 4D cone beam CT: first experimental results. Phys Med Biol. 2006; 51 (11): 2939–2952. 16723776 10.1088/0031-9155/51/11/017

[12] Sillanpaa J , Chang J , Mageras G , et al. Developments in megavoltage cone beam CT with an amorphous silicon EPID: reduction of exposure and synchronization with respiratory gating. Med Phys. 2005; 32 (3): 819–929. 15839355 10.1118/1.1861522

[13] Chang J , Sillanpaa J , Ling CC , et al. Integrating respiratory gating into a megavoltage cone‐beam CT system. Med Phys. 2006; 33 (9): 2354–2361. 16898437 10.1118/1.2207136

[14] Pang G , Rowlands JA . Just‐in‐time tomography (JiTT): a new concept for image‐guided radiation therapy. Phys Med Biol. 2005; 50 (21): N323–N330. 16237232 10.1088/0031-9155/50/21/N05

[15] Siewerdsen JH , Jaffray DA . A ghost story: spatio‐temporal response characteristics of an indirect‐detection flat‐panel imager. Med Phys. 1999; 26 (8): 1624–1641. 10501063 10.1118/1.598657

[16] Siewerdsen JH , Jaffray DA . Cone‐beam computed tomography with a flat‐panel imager: effects of image lag. Med Phys. 1999; 26 (12): 2635–2647. 10619249 10.1118/1.598803

[17] McDermott LN , Nijsten SM , Sonke JJ , Partridge M , van Herk M , Mijnheer BJ . Comparison of ghosting effects for three commercial a‐Si EPIDs. Med Phys. 2006; 33 (7): 2448–2451. 16898447 10.1118/1.2207318

[18] Pang G , Lee DL , Rowlands JA . Investigation of a direct conversion flat panel imager for portal imaging. Med Phys. 2001; 28 (10): 2121–2128. 11695774 10.1118/1.1405844

[19] Zhao W , DeCrescenzo G , Kasap SO , Rowlands JA . Ghosting caused by bulk charge trapping in direct conversion flat‐panel detectors using amorphous selenium. Med Phys. 2005; 32 (2): 488–500. 15789596 10.1118/1.1843353

[20] Schroeder C , Stanescu T , Rathee S , Fallone BG . Lag measurement in an a‐Se active matrix flat‐panel imager. Med Phys. 2004; 31 (5): 1203–1209. 15191310 10.1118/1.1713298

[21] Herman MG , Balter JM , Jaffray DA , et al. Clinical use of electronic portal imaging: report of AAPM Radiation Therapy Committee Task Group 58. Med Phys. 2001; 28 (5): 712–737. 11393467 10.1118/1.1368128

[22] Antonuk LE , Boudry J , Huang W , et al. Demonstration of megavoltage and diagnostic X‐ray imaging with hydrogenated amorphous silicon arrays. Med Phys. 1992; 19 (3): 1455–1466. 1461210 10.1118/1.596802

[23] Munro P , Bouius DC . X‐Ray quantum limited portal imaging using amorphous silicon flat‐panel arrays. Med Phys. 1998; 25 (5): 689–702. 9608480 10.1118/1.598252

